# Latvian Primary Care Management of Children with Acute Infections: Antibiotic-Prescribing Habits and Diagnostic Process Prior to Treatment

**DOI:** 10.3390/medicina57080831

**Published:** 2021-08-17

**Authors:** Zane Likopa, Anda Kivite-Urtane, Jana Pavare

**Affiliations:** 1 Children’s Clinical University Hospital, Vienibas Gatve 45, LV-1004 Riga, Latvia; jana.pavare@rsu.lv; 2 Department of Public Health and Epidemiology, Institute of Public Health, Riga Stradins University, Kronvalda Bulvaris 9, LV-1010 Riga, Latvia; Anda.kivite-urtane@rsu.lv; 3 Department of Pediatrics, Riga Stradins University, Vienibas Gatve 45, LV-1004 Riga, Latvia

**Keywords:** acute infections, children, antibiotic prescription, primary care, diagnostic test

## Abstract

*Background and Objectives:* Primary care physicians frequently prescribe antibiotics for acutely ill children, even though they usually have self-limiting diseases of viral etiology. The aim of this research was to evaluate the routine antibiotic-prescribing habits of primary care in Latvia, in response to children presenting with infections. *Materials and Methods:* This cross-sectional study included acutely ill children who consulted eighty family physicians (FP) in Latvia, between November 2019 and May 2020. The data regarding patient demographics, diagnoses treated with antibiotics, the choice of antibiotics and the use of diagnostic tests were collected. *Results:* The study population comprised 2383 patients aged between one month and 17 years, presenting an acute infection episode, who had a face-to-face consultation with an FP. Overall, 29.2% of these patients received an antibiotic prescription. The diagnoses most often treated with antibiotics were otitis (45.8% of all antibiotic prescriptions), acute bronchitis (25.0%) and the common cold (14.8%). The most commonly prescribed antibiotics were amoxicillin (55.9% of prescriptions), amoxicillin/clavulanate (18.1%) and clarithromycin (11.8%). Diagnostic tests were carried out for 59.6% of children presenting with acute infections and preceded 66.4% of antibiotic prescriptions. *Conclusion*: Our data revealed that a high level of antibiotic prescribing for self-limiting viral infections in children continues to occur. The underuse of narrow-spectrum antibiotics and suboptimal use of diagnostic tests before treatment decision-making were also identified. To achieve a more rational use of antibiotics in primary care for children with a fever, professionals and parents need to be better educated on this subject, and diagnostic tests should be used more extensively, including the implementation of daily point-of-care testing.

## 1. Introduction

Acute illness in pediatric patients is a common reason for seeking the help of family physicians (FP). However, in these circumstances, the children mostly have self-limiting viral infections that do not require specific treatment.

Antibiotics are the most commonly administered prescription drugs for children [1], around 90% of which are prescribed in primary care. Studies in European countries have shown that more than 50% of children have received at least one course of antimicrobial therapy before the age of one [2,3]. Of particular concern is the fact that the highest antibiotic prescription rates are in the age group from two to five years old, as we know that in this age group, viral infections dominate [4], and the use of broad-spectrum antibiotics rather than narrow-spectrum ones is increasing [5]. A recent study has also shown that broad-spectrum antibiotics are often prescribed as the first-choice therapy in Latvia, thus contributing to the growing resistance to antibiotics [6].

In pediatric populations, the most common reason for antibacterial treatment is upper and lower respiratory tract infections [7]. However, despite the evidence showing little or no benefit from antibiotic therapies, up to 80% of patients consulting primary care clinicians for these complaints are still prescribed them [8,9].

Several factors contribute to the unwarranted and extensive use of antimicrobials in children in primary care: incomplete clinical evaluation of children with a fever; a lack of evidence-based decisions on the part of doctors; diagnostic uncertainty in differentiating viral and bacterial infections, and a fear of missing serious bacterial infections; an extensive phobia of fever amongst parents [9,10]. These factors also lead to unnecessary hospital referrals, needless additional testing, and an increase in the number of potentially avoidable short-stay hospital admissions for children [3].

Point-of-care testing (POCT), defined as medical diagnostic testing at or near the site of patient care, is a fast and simple tool that can support clinical decision-making and improve the quality of primary care for children [3]. For acute illnesses, POCT such as group A streptococcal antigens, C-reactive protein level, urine strips and bacteriological cultures may be useful and may suppress rushed antibiotic prescribing [8]. Therefore, the usage of diagnostic tests has the potential to improve targeted antimicrobial treatment; however, in Latvia, POCT usage before decision-making has been reported to be very low [6].

The unwarranted use of antibacterial therapies contributes to avoidable adverse effects, healthcare costs and the emergence of antibiotic resistance. Growing antimicrobial resistance is a major problem in global healthcare and, consequently, a significant rationalization and reduction of antibiotic use is essential [5,11].

The aim of this research was to evaluate the routine antibiotic-prescribing habits of primary care in Latvia, in response to children presenting with infections.

## 2. Materials and Methods

The cross-sectional study was conducted in Latvia between November 2019 and May 2020. In order to evaluate the management of infectious diseases and the routine prescribing of antibiotics for children in primary care in Latvia, 80 FP from various Latvian regions were asked to record data on pediatric patients, aged from 1 month to 17 years, with an acute infection episode, who attended consultations during face-to-face appointments. There are about 360,000 children in Latvia and they are cared for by about 1300 FP, who are self-employed and are usually located independently but, apart from these practitioners, there are also 22 primary-care pediatricians.

### 2.1. Participating Children

Inclusion criteria:

- Current clinical signs of acute infection for less than five days;
- Aged one month up to 17 years old;
- Exclusion criteria:
- Aged under 1 month;
- Re-convalescent stage of infectious disease;
- Use of antimicrobial therapy before the time of the visit.

### 2.2. Sample Size Calculation

Table 1 details the sample-size calculation. The target group of the study is the underage population of Latvia (0–17 years of age), visiting their FP due to an infectious disease. The sampling method of the current study was according to their place of residence and age-stratified convenience sampling, recruited via FPs. The sample size (column (f)) was calculated, taking into consideration the following criteria:

1. The total number of children aged 0–17 in 2019 (column (b)), according to the population registry of Latvia [12];
2. The proportion of children visiting FP within a year because of infectious diseases is 39% in children aged 0–4 years, and 8% in older ones [13] (respective calculated size of the target population—column (c));
3. The time schedule of the research is 6 months (respective calculated size of the target population—column (d));
4. The proportion of children receiving an antibiotic prescription in the case of an infectious disease is on average 56% in the age group of 0–4 years, 23% in the age group of 5–9 years, and 22% in the age groups of 10–14 years and 15–17 years [4,14,15] (column (e));
5. The chosen confidence limit is ±5%.

**Table 1:** Size of the target population and the study sample.

Strata (a)		Number of Inhabitants, Latvia (b)	Visiting FP within a Year (c)	Visiting FP within 6 Months (d)	Frequency of AB Prescriptions (%) (e)	Sample Size (f)
0–4 years	Urban	56,072	21,868	10,934	56	366
	Rural	70,412	27,461	13,730	56	369
5–9 years	Urban	50,574	4046	2023	23	240
	Rural	66,470	5318	2659	23	247
10–14 years	Urban	50,408	4033	2016	22	234
	Rural	69,916	5593	2797	22	242
15–17 years	Urban	26,225	2098	1049	22	211
	Rural	38,128	3050	1525	22	225
					Total:	2134

Abbreviations: FP—family physician; AB—antibiotic.

### 2.3. Participating Family Physicians

The target group of the participating doctors was 1268 FP from the Latvian register of family physicians. It was expected that each FP might see about 30 suitable patients during the study. According to the sample size of 2134 patients, a total of 80 FP was required. The participating doctors were recruited using two approaches. First, from the 1268 FPs, through an Excel random-number generator, we selected 160 doctors (the expected response rate was 50%) across different geographically located practices (urban and rural areas) and sent invitations in both email and letter form to participate in the study. The response rate was lower than expected, and we recruited only 38 participants using this approach. Secondly, we directly addressed doctors at a meeting of the Latvian Family Physicians Association and achieved the requisite number of 80 participants. Of these 80 FP, 34 were located in the capital of Latvia, which was in proportion to the distribution of the population within the country. The participating doctors were regularly practicing physicians who were not normally involved in academic research. Doctors who included fewer than five patients were excluded from the study. The data from 73 practices were analyzed.

### 2.4. Data Collection

The data were collected in anonymized form, including patient demographics, diagnoses based on a pre-defined list, laboratory tests performed before the initiation of antimicrobial treatment (such as a full blood count, C-reactive protein measurement from venous blood samples or POCT from capillary blood, urine test strips and microscopy, group A streptococcal rapid antigen testing, rapid influenza diagnostic tests, bacteriological cultures, X-ray) and—in the case of antibiotic prescription—whether it was a delayed or immediate antibiotic prescription and choice of antimicrobial group.

### 2.5. Statistical Analyses

Descriptive statistics, such as means (with standard deviations) and medians for continuous variables, and the proportions for categorical variables, were calculated. The Kolmogorov–Smirnov test was used to test the normality of distribution. To evaluate the statistical significance of the differences in proportions of dependent variables between subgroups of independent variables, either the chi-square test or Fisher’s exact test was used. Statistical significance was set at *p* = 0.05. Data processing was performed using IBM SPSS Statistics (Statistical Package for the Social Sciences, Version 23.0).

The main outcome measure was antibiotic prescribing and the type of antibiotics.

The study was approved by the Ethics Committee of Riga Stradins University, approval no. 6-3/5/21 (30 May 2019).

## 3. Results

**Figure 1:**
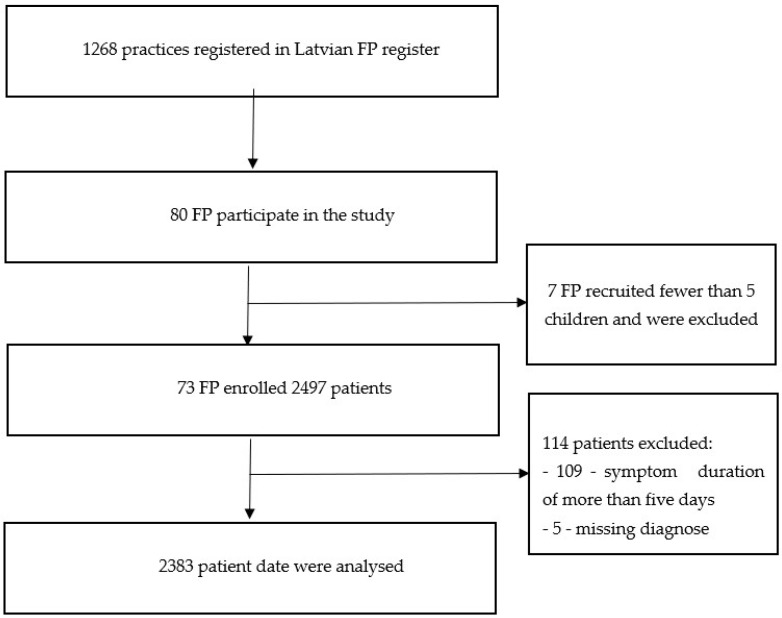
Flowchart of included family physicians (FP) and recruited illness episodes.

During the six-month study period, 2497 children with acute illnesses were enrolled. In total, 2383 patients met the inclusion criteria, after 109 patients were excluded due to a symptom duration of more than five days, and 5 patients were excluded because of missing diagnoses (Figure 1).

The mean number of included patients per FP was 29.8. The mean age was 6.1 years, and the median, 5.0 years. Boys comprised 50.1% of the study participants. Table 2 details the characteristics of the studied population.

**Figure 2:**
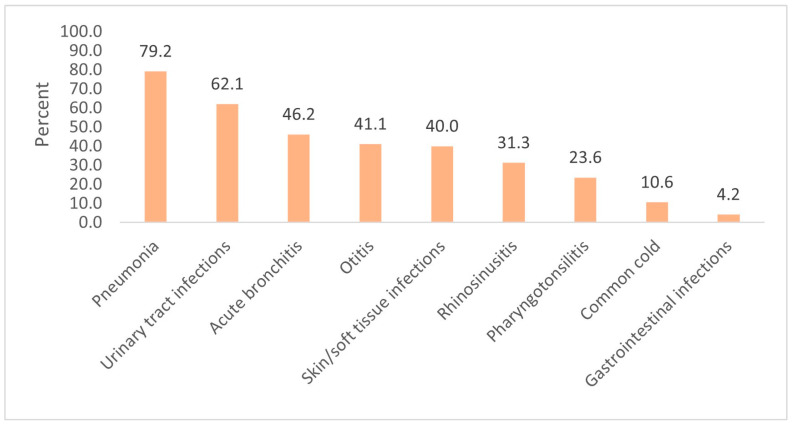
The proportion of patients (%) treated with antibiotics for each type of infection.

According to the physicians’ diagnoses, the most common infections observed were the common cold (40.8%), otitis (32.6%), and acute bronchitis (15.8%). These diagnoses were the ones most often treated with antibiotics; otitis was responsible for 45.8% of all antibiotic prescriptions, acute bronchitis for 25.0%, and the common cold for 14.8%. Table 3 lists all the diagnoses observed, the number of patients diagnosed with each type of infection, and the number of patients receiving an antibiotic prescription (the distribution of diagnoses treated with antibiotics is shown in parentheses). The proportion of patients treated with antibiotics for each type of infection is shown in Figure 2.

Overall, 29.2% of patients received an antibiotic prescription—554 ambulatory visits (23.2%) prompted immediate antibiotic prescriptions, 132 patients (5.5%) received delayed antibiotic prescriptions, while, for 11 patients (0.5%), antibiotic prescriptions were related to social indications. The mean age of the children who were prescribed an antibiotic was 5.8 years, the median 4.0 years. In all, 31.4% of patients in the age group of 0–4 years received an antibiotic prescription, 28.0% in the age group of 5–9 years, 22.9% in the age group of 10–14 years, and 34.3% in the age group of 15–17 years. The proportions of antibiotic prescriptions in the different age groups were significantly different (*p* = 0.008), particularly between the age groups of 0–4 years and 10–14 years (*p* = 0.002), and between 10–14 years and 15–17 years (*p* = 0.009).

Antibiotics were prescribed for 29.8% of the girls and for 28.8% of the boys. This difference between the genders was not statistically significant (*p* = 0.59).

In total, 63.4% of patients were consulted on the second and third days of illness, and more than half (61.6%) of antibiotic prescriptions occurred in this symptom duration range. 278 patients’ data were missing information on the duration of symptoms.

The proportions of antibiotic prescriptions in relation to the duration of symptoms are presented in Table 4. The proportions of antibiotic prescriptions in the different durations of symptoms groups were significantly different (*p* = 0.04), particularly between the 1st day and the 3rd day (*p* = 0.047), the 2nd day and the 3rd day (*p* = 0.004), the 2nd day and the 4th day (*p* = 0.01) and the 2nd day and the 5th day (*p* = 0.02).

In total, 12 different antibiotics were prescribed. In almost all cases, the drugs were administered per os. However, two patients received antibiotics intravenously after being sent to the hospital, following their examination in primary care (one patient received ampicillin J01CA01, and the other received ceftriaxone).

Penicillins represented 80.3% of all prescriptions for antibiotics and were the most widely used drug in all the age groups. Specifically, 55.9% of administered penicillins were extended-spectrum types, 18.1% were in combination with beta-lactamase inhibitors, while just 6.1% were narrow-spectrum beta-lactamase-sensitive penicillins. Penicillins were mostly prescribed for cases of acute otitis (50.6%), acute bronchitis (25.0%) and the common cold (15.0%). Penicillins were followed by macrolides 12.6% (37.1% for pneumonia, 32.6% for acute bronchitis and 18.0% for otitis) and cephalosporins 4.1% (44.8% for otitis, 17.2% for acute bronchitis and 17.2% for pneumonia). Of the cephalosporins, 93.1% were second-generation ones. Overall, the most commonly prescribed antibiotics were amoxicillin (55.9% of prescriptions), amoxicillin/clavulanate (18.1%) and clarithromycin (11.8%).

**Figure 3:**
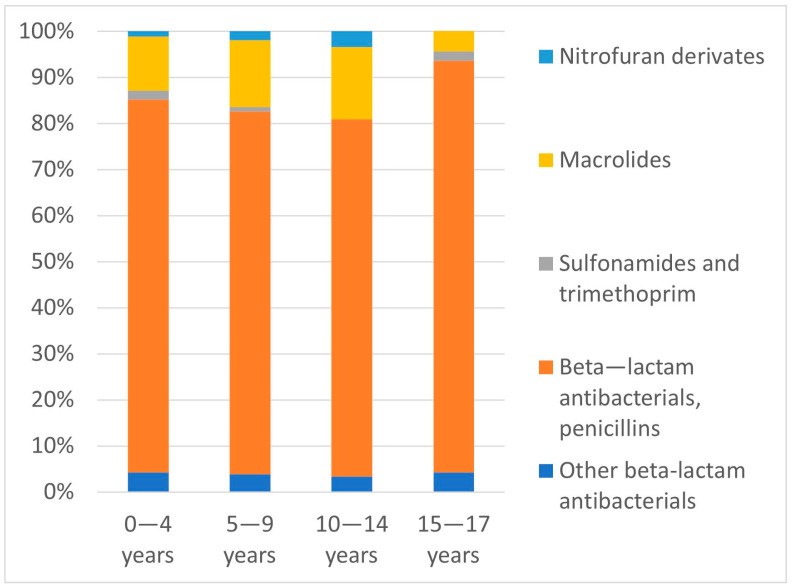
Proportions of prescribed antibiotic subgroups (%) relative to patient age groups.

Figure 3 illustrates the most common antibiotic groups according to patient age. The proportions of prescribed antibiotic subgroups in the different age groups were not significantly different (*p* = 0.57).

**Figure 4:**
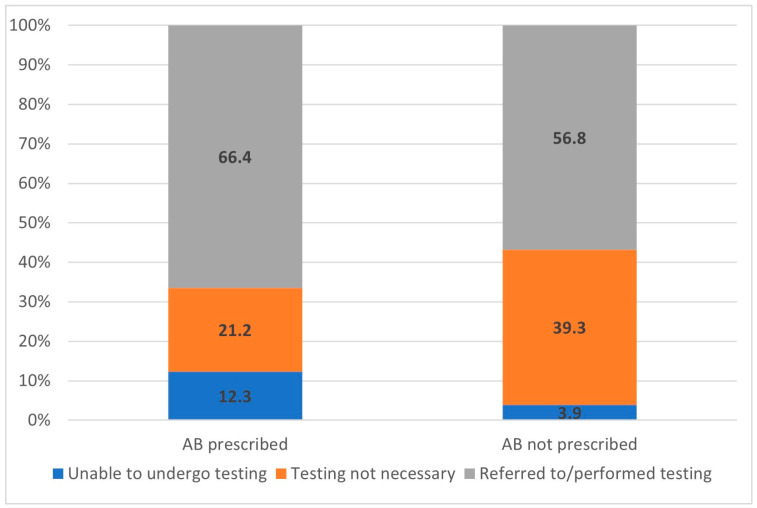
Proportions of diagnostic management options (%) before a decision of antibiotic (AB) prescribing is made, according to the groups of antibiotic prescription approaches.

Laboratory or radiological investigations were carried out for 59.6% of the study population and for 66.4% of the cases treated with antibiotics. However, all other decisions of antibiotic prescribing were based only on clinical assessment, either due to the unavailability of testing (12.3%) or because investigations were deemed to be unnecessary, according to the doctor’s opinion (21.2%). The proportion of diagnostic testing prior to antibiotic prescribing is indicated in Figure 4.

The frequency of diagnostic testing significantly increased as the duration of symptoms at the time of the visit increased (*p* < 0.001): 27.2% of patients were referred for testing on the first day of illness; 52.1% on the second day; 60.2% on the third day; 66.4% on the fourth day; 71.4% on the fifth day.

By far, the most frequently performed test was the measurement of the level of C-reactive protein; 53.0% (*n* = 1262) of children presenting for medical visits with acute infections; and 56.0% of patients who received antimicrobial treatment. This test was performed at a significantly higher frequency in older children (*p* < 0.001): 70.7% in the age group of 15–17 years; 57.1% in the age group of 10–14 years; 54.4% in the age group of 5–9 years; 48.0% in the age group of 0–4 years.

Although 23.6% (*n* = 20) of patients with pharyngotonsillitis were treated with antibiotics, only 2.8% (*n* = 2) of patients with this diagnosis were tested for group A streptococci. X-ray imaging was used for 45.8% (*n* = 33) of patients with pneumonia, while dipstick urine tests or urine analyses were used for 72.4% (*n* = 21) of patients with urinary tract infections.

## 4. Discussion

The purpose of this study was to determine the antibiotic-prescribing habits of primary care in Latvia. Importantly, this is the first time that pediatric patients have been exclusively analyzed. Studies on outpatient antibiotic treatment practices in Latvia have previously been published by Dumpis et al. (2013 data from Latvia only [16], and 2018 data from Latvia, Lithuania and Sweden, were compared [6]). However, these studies had patient populations that included both children and adults.

Our data showed that 29.2% of pediatric patients with an acute illness episode, consulting primary care, received an antibiotic prescription. This prescription rate is lower than the ones reported by Dumpis et al. (42% in Latvia and Lithuania, 38% in Sweden). Moreover, patients younger than 20 years of age comprised the majority of all patients receiving antibiotics in Latvia (51%) and Lithuania (53%), while in Sweden this age group comprised 33% [6].

We found that 5.5% of patients received a delayed antibiotic prescription. This is one of the strategies to try and reduce antibiotic use for respiratory infections, and has been reported to engender similar patient satisfaction to immediate antibiotic prescription [17]. However, many patients suffering from the common cold still expect to be prescribed an antibiotic when visiting their FP [18].

The highest antibiotic prescription rates were observed in the age groups of 0–4 years (31.4%) and 15–17 years (34.3%). Studies in other countries have shown a decline in the prescription rate with increasing age, and that one-year-olds [2] and two- to five-year-olds [4] had the highest rate of antibiotic use. In Germany and the Netherlands, children in the 2–5 years age group and those older than 15 years were found to receive antibiotics most often [18,19] 

In this study, in order to focus on the early initiation of antibacterial treatment, only patients with a symptom duration of under five days were analyzed. Dumpis et al.’s 2018 study analyzed patients with longer symptom duration—6.4 days in Latvia, 7.3 days in Lithuania, and 11 days in Sweden. This suggests that, in Sweden, patients with viral infections tend to be given symptomatic treatment at home for a longer period [6]. Almost 70% of our patients were consulted within the first three days of the onset of symptoms, and a substantial amount of antibiotics was prescribed at the beginning of the illness episodes. This trend for the early initiation of antibacterial treatment may be due to several factors that have previously been described: difficulties differentiating viral from bacterial infections [20]; overdiagnosis of certain conditions [21]; fear of missing serious bacterial infections; parental insistence on antibiotics [22]. Interestingly, in low-prescribing regions, there are no data that suggest worse outcomes for acute illness episodes due to undertreatment [21].

### 4.1. Diagnoses Treated with Antibiotics

In line with other studies, we found that respiratory infections were the main indicator for visiting the FP during acute illness episodes [16,23]. Although it is well known that acute respiratory infections are predominantly of viral etiology and are self-limiting, in countries other than Latvia, this type of infection also accounts for the majority of antibiotic prescriptions [24].

Otitis, acute bronchitis and the common cold were the most common diagnoses treated with antibiotics, responsible for about 85% of all antibiotics prescribed. Our data are in accordance with data collected in Germany and the Netherlands [18] where the most frequent indications for antibiotic prescriptions for children were otitis media, tonsillitis, other upper respiratory tract infections, and bronchitis (70–80% of antibiotic prescriptions). A number of recent systematic reviews suggest that antibiotics only slightly modify the course of otitis media, tonsillitis and bronchitis, and have no effect on the course of the common cold [17].

We found a low antibiotic prescription rate for otitis (41.1%) compared with other studies, for example, 56% in the Netherlands [25], and more than 90% in Ireland [23] and the United States [26]. Additionally, we observed an antibiotic prescription rate of 46.2% for acute bronchitis, whereas a lower rate has been reported in Sweden (21%) and higher rates in Lithuania (68%), Latvia (72%; as published in Dumpis et al.’s 2018 study [6]), and Ireland (84%) [23]. However, it should be noted that the pediatric population was not analyzed separately in these studies. A relatively small number of patients who consulted their FP received a diagnosis of pharyngotonsillitis in our study (*n* = 72, 3.0% of all episodes); the prescription rate (23.6%) was lower than in other studies (53.11% for patients with a sore throat and 94.87% with tonsillitis) [23].

### 4.2. Choice of Antibiotic

Broad-spectrum antibiotics are still being widely used in primary care [4]. In this study, by far the most prescribed antibiotic class used in all four age groups was penicillin, accounting for 80% of all antibiotics prescribed. This finding complies with the majority of recommendations to use penicillin as the first-line therapy for most common pediatric respiratory infections. Similar findings have been described in primary care in the Netherlands [7]. Broad-spectrum penicillins accounted for the majority of penicillin prescriptions, while just 6.1% were narrow-spectrum ones. This was also the case in an earlier study conducted in Latvia [16], and this may be due to the limited availability of phenoxymethylpenicillin in the country and/or, in contrast to other penicillins, its non-inclusion in the list of reimbursed drugs for children.

Macrolides were the second most frequently prescribed antibiotics (12.6%). In agreement with previous studies [4], they were utilized to the greatest extent in the age group of 10–14 years (15.7%); however, no significant differences in their percentage usage were observed among the four age groups.

In contrast to the relatively high use of macrolides, cephalosporins accounted for only 4% of prescriptions. Our data are consistent with those reported in 2018 by Dumpis et al. [6]. It has been observed that cephalosporins are the second most frequently consumed class of antibiotics in Germany, with cefuroxime being the most often prescribed of this class, whereas they have been noted to be very rarely used in the Netherlands, the United Kingdom and Scandinavian countries [4,18].

The three most often used drugs in the study were amoxicillin, amoxicillin/clavulanate and clarithromycin. Amoxicillin has been reported to be the leading antibiotic prescribed in the Netherlands, Germany and Canada, with amoxicillin/clavulanate being the most prescribed one in Italy and Ireland [18,23] and the second most frequently prescribed one in the Netherlands [7]. Clarithromycin is widely used in many countries in Europe; however, it belongs to the 2019 WHO AWaRe classification database’s “watch group” of antibiotics, which have higher resistance potential.

### 4.3. Diagnostic Management before Antibiotic Prescribing

FP utilized diagnostic tools for only 66.4% of patients before prescribing antibiotics. Accurate diagnostic testing has the potential to support clinical decision-making, reduce the overtreatment of viral infections, and provide reassurance to physicians regarding postponing immediate antibacterial treatment [20]. In 12.3% of instances of antibiotic prescribing, the FP was uncertain of the nature of the patient’s illness and considered it necessary to carry out additional diagnostic tests before deciding on their treatment. However, this was not possible, as the relevant tests were not available on the day of the patient’s visit. It is likely that this situation increases the rate of empirical antibacterial treatment.

Measurement of the level of C-reactive protein was the most frequently performed diagnostic test in our study (53.0% of patients). This is in line with data from Sweden, showing that this test is performed for about 50% of patients with respiratory infections. In most Scandinavian countries, Germany, and Switzerland, it is widely available as a POCT [27,28]. However, in Latvia, a POCT of the level of C-reactive protein is available in only a few medical practices, and so most of the analyses requested by FP continue to be conducted in central laboratories. This situation hinders clinical decision-making, as the FP receives the test results after their patient’s visit, possibly leading to overtreatment or extra visits. POCT in ambulatory care has other benefits over laboratory testing: it is easy to use and more child-friendly, as a finger prick-test is less invasive than a venous puncture; it provides timely results, allowing treatment to be appropriately adapted during the patient’s visit and giving the FP more confidence to withhold unnecessary treatment (which, in turn, manages parents’ expectations for antibiotics and improves their satisfaction) [3,29,30,31,32].

With respect to respiratory infections, the combination of POCT of the level of C-reactive protein and clinical assessment has been shown to have a significant effect on antibiotic prescribing. For instance, an approximately 30% reduction in the prescription rate has been reported. However, the data have mostly been derived from adult populations [28], and the value of testing for the management of children with acute infections remains unconvincing [3]. Nevertheless, promising results have been seen in studies where the usage of POCT for C-reactive protein levels and training in communications skills [9], safety-net advice for parents [20], and FP educational interventions, were combined [33].

It is important to point out that this study has several limitations. The FP response rate was lower than expected in random selection, and FPs included in the study may have been more active and willing to avoid antibiotic prescribing. Patient inclusion was distributed over a six-month period, and was not inclusive of all patients visiting their FP. We did not reach the sample size for children older than 10 years, as they visited their FP less often due to respiratory infections and were often at a later stage of the disease [19]. The rate of patient recruitment was slower than anticipated, due to a reduced number of acute illness episodes in FP practices in the spring of 2020, as a consequence of SARS-CoV-2 virus (COVID-19) epidemiological safety measures. One strength of our study is the involvement of various FP practices from different geographical locations and the availability of laboratory testing, which could potentially be found to have an impact on antibiotic-prescribing habits.

## 5. Conclusions

Our results demonstrate that a high level of antibiotic prescribing for self-limiting viral infections in children still exists, that narrow-spectrum antibiotics are underused, and that there is a suboptimal use of diagnostic tests prior to treatment decision-making.

To achieve a more rational use of antibiotics in primary care for children with a fever, professionals and parents need to be better educated on this subject, and diagnostic tests should be used more extensively, including a greater implementation of daily point-of-care testing.

## Figures and Tables

**Table 2:** Characteristics of the studied population and those patients treated with antibiotics.

Variable	Study Population
Mean age (all patients, years)	6.1 (SD 4.3)
Age groups	
0–4 years	1094 (45.9%)
5–9 years	743 (31.2%)
10–14 years	371 (15.6%)
15–17 years	140 (5.9%)
Missing information on age	35 (1.5%)
Gender	
Female	1168 (49.0%)
Male	1195 (50.1%)
Missing information on gender	20 (0.8%)
Patients receiving an antibiotic prescription	697 (29.2%)
Mean age of patients who received an antibiotic (years)	5.8 (SD 4.4)

**Table 3:** Major types of infections for all recruited patients and patients treated with antibiotics.

Diagnosis	Number of All Patients	Number of Patients Receiving an Antibiotic Prescription
Common cold	973 (40.8%)	103 (14.8%)
Otitis	776 (32.6%)	319 (45.8%)
Rhinosinusitis	16 (0.7%)	5 (0.7%)
Pharyngotonsillitis	72 (3.0%)	17 (2.4%)
Other otorhinolaryngological (ORL) infection	14 (0.6%)	0 (0.0%)
Acute bronchitis	377 (15.8%)	174 (25.0%)
Pneumonia	72 (3.0%)	57 (8.2%)
Gastrointestinal infections	48 (2.0%)	2 (0.3%)
Urinary tract infections	29 (1.2%)	18 (2.6%)
Skin/soft tissue infections	5 (0.2%)	2 (0.3%)
Bone and joint infections	1 (0.0%)	0 (0.0%)
Total	2383 (100%)	697 (100%)

**Table 4:** Proportions of antibiotic (AB) prescriptions, relative to the duration of symptoms.

Duration of Symptoms before Visit (Days)	AB Prescribed	AB Not Prescribed	Total
1	23 (22.3%)	80 (77.7%)	103 (100.0%)
2	159 (24.8%)	482 (75.2%)	641 (100.0%)
3	222 (32.0%)	472 (68.0%)	694 (100.0%)
4	140 (31.8%)	300 (68.2%)	440 (100.0%)
5	74 (32.6%)	153 (67.4%)	227 (100.0%)
Total	618 (29.4%)	1487 (70.6%)	2105 (100%)

## Data Availability

The data presented in this study are available on request from the corresponding author.
